# Somatic *MED12* mutations in uterine leiomyosarcoma and colorectal cancer

**DOI:** 10.1038/bjc.2012.428

**Published:** 2012-09-20

**Authors:** K Kämpjärvi, N Mäkinen, O Kilpivaara, J Arola, H-R Heinonen, J Böhm, O Abdel-Wahab, H J Lehtonen, L M Pelttari, M Mehine, H Schrewe, H Nevanlinna, R L Levine, P Hokland, T Böhling, J-P Mecklin, R Bützow, L A Aaltonen, P Vahteristo

**Affiliations:** 1 Department of Medical Genetics, Genome-Scale Biology Research Program, University of Helsinki, PO Box 63, Helsinki FIN-00014, Finland; 2 Department of Pathology, The Laboratory of Helsinki University Central Hospital (HUSLAB), Helsinki University Central Hospital and Haartman Institute, University of Helsinki, PO Box 21, Helsinki FIN-00014, Finland; 3 Department of Pathology, Jyväskylä Central Hospital, Keskussairaalantie 19, Jyväskylä FIN-40620, Finland; 4 Human Oncology and Pathogenesis Program, Memorial Sloan-Kettering Cancer Center, 1275 York Avenue, Box 20, New York, NY 10065, USA; 5 Department of Obstetrics and Gynecology, University of Helsinki and Helsinki University Central Hospital, PO Box 700, Helsinki FIN-00029, Finland; 6 Department of Developmental Genetics, Max Planck Institute for Molecular Genetics, Ihnestrasse 73, Berlin 14195, Germany; 7 Department of Hematology, Aarhus University Hospital, Tage-Hansens Gade 2, Aarhus C DK-8000, Denmark; 8 Department of Surgery, Jyväskylä Central Hospital and University of Eastern Finland, Keskussairaalantie 19, Jyväskylä FIN-40620, Finland

**Keywords:** *MED12*, mutation screening, somatic mutation, benign tumours, malignant tumours

## Abstract

**Background:**

Mediator complex participates in transcriptional regulation by connecting regulatory DNA sequences to the RNA polymerase II initiation complex. Recently, we discovered through exome sequencing that as many as 70% of uterine leiomyomas harbour specific mutations in exon 2 of *mediator complex subunit 12* (*MED12*). In this work, we examined the role of *MED12* exon 2 mutations in other tumour types.

**Methods:**

The frequency of *MED12* exon 2 mutations was analysed in altogether 1158 tumours by direct sequencing. The tumour spectrum included mesenchymal tumours (extrauterine leiomyomas, endometrial polyps, lipomas, uterine leiomyosarcomas, other sarcomas, gastro-intestinal stromal tumours), hormone-dependent tumours (breast and ovarian cancers), haematological malignancies (acute myeloid leukaemias, acute lymphoid leukaemias, myeloproliferative neoplasms), and tumours associated with abnormal Wnt-signalling (colorectal cancers (CRC)).

**Results:**

Five somatic alterations were observed: three in uterine leiomyosarcomas (3/41, 7% Gly44Ser, Ala38_Leu39ins7, Glu35_Leu36delinsVal), and two in CRC (2/392, 0.5% Gly44Cys, Ala67Val).

**Conclusion:**

Somatic *MED12* exon 2 mutations were observed in uterine leiomyosarcomas, suggesting that a subgroup of these malignant tumours may develop from a leiomyoma precursor. Mutations in CRC samples indicate that *MED12* may, albeit rarely, contribute to CRC tumorigenesis.

Mediator is a large multiprotein complex, which is involved in global as well as gene-specific transcriptional regulation of most protein coding genes. The complex can both activate and repress transcription by connecting transcription factors to the RNA polymerase II initiation complex (Taatjes, 2010). *Mediator complex subunit 12* (*MED12*) gene on Xq13.1 encodes MED12 protein, which together with MED13, CDK8, and Cyclin C comprises a CDK8 submodule of the Mediator. This submodule participates in transcriptional regulation, as well as in scaffold formation and transcription elongation (Galbraith *et al*, 2010). Mediator complex subunit 12 is an essential regulator of the kinase activity of CDK8 submodule, and the protein directly interacts with various transcription factors (Knuesel *et al*, 2009; Taatjes, 2010). Mediator complex subunit 12 participates in various molecular pathways, for example, p53 and Wnt/*β*-catenin pathways, which have central roles in tumour development (Kim *et al*, 2006; Galbraith *et al*, 2010).

Uterine leiomyomas, also known as fibroids, are benign smooth muscle tumours that occur in approximately 70% of women by the age of 50 years (Day Baird *et al*, 2003). Despite their benign nature, these tumours cause various symptoms including abdominal pain, abnormal menstrual bleeding, pregnancy complications, and even infertility. Oestrogen and progesterone dependency is a characteristic feature for uterine leiomyomas, which usually occur in women of reproductive age and typically regress after menopause (Parker, 2007). Several recurrent, albeit infrequent, genetic aberrations have been observed in these tumours, including deletions in chromosome 7q, trisomy of chromosome 12, various rearrangements affecting the *high mobility group AT-hook 2* (*HMGA2*) gene, and structural changes at 6p21 (Mitelman, 1998). Recently, we discovered by exome sequencing that as many as 70% of uterine leiomyomas harbour very specific somatic mutations in *MED12* (Mäkinen *et al*, 2011a). Strikingly, all mutations resided in exon 2, and the vast majority of them affected a single codon glutamine 44. The finding has subsequently been validated in other populations (Mäkinen *et al*, 2011b; Je *et al*, 2012; Markowski *et al*, 2012; McGuire *et al*, 2012). *Mediator complex subunit 12* had not been implicated in human tumorigenesis before identification of specific exon 2 mutations in uterine leiomyomas. High mutation frequency and the proteins’ key role in transcriptional regulation prompted us to study the genes role in other tumour types. We collected a comprehensive series of 1158 samples representing various tumour types that might harbour mutations in the gene. These include both benign and malignant mesenchymal tumours, oestrogen- and progesterone-dependent tumours, and haematological malignancies, as retroviral insertions in *Med12* have been reported to participate in the development of leukaemias in murines (Dave *et al*, 2009). We also included tumours associated with abnormal Wnt-signalling, as preliminary data indicated this well-known cancer-related pathway may be dysregulated in *MED12* mutation-positive uterine leiomyomas (Mäkinen *et al*, 2011a).

## Materials and Methods

### Subjects

Altogether 1158 tumours from as many patients were included in the study. The sample series consisted of 286 mesenchymal tumours (uterine leiomyosarcomas, other sarcomas, gastrointestinal stromal tumours, extrauterine leiomyomas, endometrial polyps, lipomas), 216 oestrogen- and progesterone-dependent tumours (ovarian and breast carcinomas), 264 haematological malignancies (acute myeloid leukaemias, acute lymphoid leukaemias, and myeloproliferative neoplasms), and 392 colorectal cancers (CRCs) as representatives of tumours commonly showing abnormal Wnt-signalling. See Table 1 for more details on the whole sample set utilised in the study, and Supplementary Information and Supplementary Tables S1,2 for additional information on uterine leiomyosarcomas and extrauterine leiomyomas.

The study was approved by the ethics review board of the Hospital District of Helsinki and Uusimaa (HUS), Helsinki, Finland, and the appropriate research permissions were obtained from local ethics committees.

### DNA extraction and *MED12* exon 2 mutation screening

Genomic DNA was extracted from archival formalin-fixed paraffin-embedded and fresh-frozen tissue samples with standard methods. *Mediator complex subunit 12* exon 2 mutation status was determined by direct sequencing. Two different sets of primers were used in the study. See Supplementary Information for more details.

## Results

Five *MED12* exon 2 mutations were identified in 1158 tumour samples, three in uterine leiomyosarcomas (3/41; 7%) and two in colorectal tumours (2/392; 0.5%) (Supplementary Table S3). No mutations were observed in any other tumour type.

Two *MED12* mutations were detected in histopathologically confirmed early-onset (dg⩽45 years) uterine leiomyosarcomas; one affecting the codon 44 that is frequently mutated in uterine leiomyomas (c.130G>A, p.Gly44Ser) and the other inserting 21 nucleotides and leading to an in-frame transcript (c.115_116ins21, p.Ala38_Leu39ins7). Corresponding normal DNA was available from these patients, and the somatic origin of the mutations was confirmed (Supplementary Figure S1). The third mutation was a three nucleotide deletion also resulting in an in-frame transcript (c.104_106delAAC, p.Glu35_Leu36delinsVal). This mutation was identified in a soft-tissue sarcoma sample, which was further diagnosed as a metastasis of uterine leiomyosarcoma. Unfortunately, normal DNA from this patient was not available for this study, and the somatic origin of this mutation could not be confirmed. It is likely, however, that this mutation is also somatic as it is located in a highly conserved area of the protein affecting the codon 36, which is the second most common mutational hotspot observed in uterine leiomyomas. Furthermore, no germline changes have been observed at this site in our own studies or reported in any published studies or in the databases.

**Figure 1:**
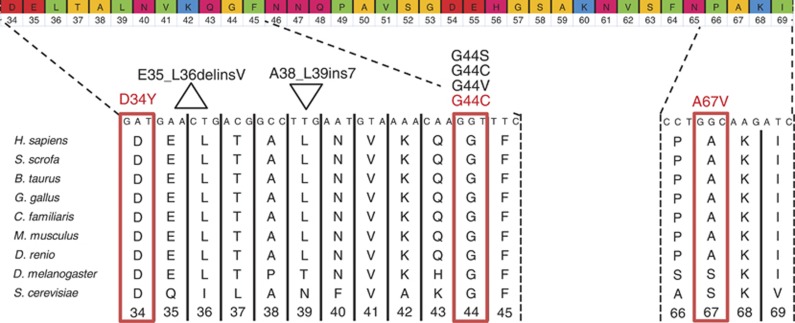
Mutations in *MED12* exon 2. The whole *MED12* exon 2 with the amino acids and codon numbers is shown at the top, and multispecies alignment of the regions with the detected mutations is shown below. Mutations observed in uterine leiomyosarcoma and CRC samples are marked with black and red, respectively. Mutations G44C and G44V in ULMS were reported by Pérot *et al* (2012). Mutation G44C in CRC was observed in this study and also in the study by The Cancer Genome Atlas Network (2012). Mutation D34Y in CRC has been reported by Je *et al* (2012). Amino acids at the top are color-coded according to their side-chains' pK_a_s (acid dissociation constant) and charge at physiological pH 7.4. Red = negatively charged, green = hydrophobic, yellow = small non-polar, magenta = polar, blue = positively charged.

The fourth mutation (c.130G>T, p.Gly44Cys), again hitting the hotspot codon 44, was identified in one CRC sample. The female patient had been diagnosed with a Dukes B/grade II/microsatellite stable tumour in the sigmoid colon at the age of 78 years. A variant with an unknown significance (c.200C>T, p.Ala67Val) was observed in one additional CRC sample. The codon is conserved among species (Figure 1), although according to *in silico* analyses with Polyphen2 (http://genetics.bwh.harvard.edu/pph2/) and SIFT (http://sift.bii.a-star.edu.sg/), this change is predicted to be tolerated. The female patient had been 78 years old when a Dukes A/grade II tumour in ascending colon was diagnosed. The tumour showed microsatellite instability. Both observed mutations in CRC samples were confirmed to be somatic (Supplementary Figure S2).

In one endometrial polyp sample, *MED12* exon 2 mutation (c.107T>G, p. Leu36Arg) was initially observed. Closer evaluation revealed that the sample also contained degenerated uterine leiomyoma tissue. Genomic DNA was extracted separately from both the polyp and leiomyoma, and the sequencing confirmed that the mutation originated from the leiomyoma.

## Discussion

Our recent study revealed very specific mutations in *MED12* exon 2 in as many as 70% of uterine leiomyomas (Mäkinen *et al*, 2011a). This study implicated, for the first time, a role for *MED12* in human tumorigenesis. To analyse whether similar mutations can be found in other tumour types, we collected a broad spectrum of samples for the *MED12* exon 2 mutation analyses.

Uterine leiomyomas originate from the smooth muscle cells and are thus of mesenchymal origin. Similar karyotypic changes that have been observed in uterine leiomyomas have also been seen in other benign mesenchymal tumours, such as extrauterine leiomyomas, endometrial polyps, and lipomas (Tallini *et al*, 2000). We therefore hypothesised that these tumours might also harbour mutations in *MED12*. We screened altogether 131 benign mesenchymal tumours, but no mutations were identified. Similar results were recently reported by Markowski *et al* (2012), who found only one mutation in a single endometrial polyp and no mutations in the lipomas. Of specific note is that none of the 42 extrauterine leiomyoma samples analysed in this study harboured *MED12* mutations.

Altogether 155 malignant mesenchymal tumours were analysed for *MED12* exon 2 mutations. Three mutations were observed, two in early-onset uterine leiomyosarcomas, and one in a metastasis of a uterine leiomyosarcoma (3/41; 7% of uterine leiomyosarcomas studied). This finding is in line with the results of a recent study by Pérot *et al* (2012), where *MED12* mutations were reported in 2/10 (20%) uterine leiomyosarcomas and 1/9 (11%) smooth muscle tumour of uncertain malignant potential, respectively. Highly aggressive and malignant uterine leiomyosarcomas are not generally considered to develop from benign leiomyomas. The observed *MED12* mutations are probably not the driving force behind the malignant transformation, but rather indicate that these tumours have developed from leiomyoma precursors. Indeed, it has also previously been suggested that a small subgroup of leiomyomas may actually develop into malignancy (Christacos *et al*, 2006). Would this be the case, identification of molecular markers that could be used in detecting such leiomyomas would be of high clinical significance as in most cases the leiomyosarcoma diagnosis is only made at surgery and many patients present with an advanced disease. In this study, no mutations were found in other sarcomas or in gastrointestinal stromal tumours. Overall, the low frequency of *MED12* exon 2 mutations in various mesenchymal tumours suggests that the high mutation frequency observed in uterine leiomyomas is not a common feature for all mesenchymal tumours.

Development and growth of uterine leiomyomas are dependent on oestrogen and progesterone. Lesions show increased expression of oestrogen and progesterone receptors, and enhanced response to oestrogen stimulation compared with normal myometrial cells has been observed. Progesterone has also been reported to increase mitotic activity and regulate cell proliferation in uterine leiomyoma cells (Parker, 2007). To elucidate whether *MED12* is involved in the tumorigenesis of other oestrogen- and progesterone-dependent tumours, we screened breast and ovarian cancer samples representing different histological subtypes for *MED12* exon 2 mutations. None of the tumours harboured mutations, indicating that aberrant hormonal function is not the underlying cause, at least alone, for *MED12* mutations in uterine leiomyomas.

Mediator complex subunit 12 interacts with *β*-catenin, and it has been identified as an important transducer of canonical Wnt/*β*-catenin signalling (Kim *et al*, 2006; Rocha *et al*, 2010). The preliminary results by us and Markowski *et al* implicate that this signalling pathway may be altered in *MED12* mutation-positive uterine leiomyomas (Mäkinen *et al*, 2011a; Markowski *et al*, 2012). Dysregulation of Wnt/*β*-catenin pathway is involved in the development of many tumour types, including CRC (Polakis, 2000). Here, we identified two *MED12* exon 2 mutations in altogether 392 CRC samples analysed. Similar results were recently reported by Je *et al* and The Cancer Genome Atlas Network, both of whom found one *MED12* exon 2 mutation in 389 (0.3%) and 224 (0.4%) CRC samples, respectively (Je *et al*, 2012; The Cancer Genome Atlas Network, 2012). Although these mutations may have occurred just by chance, these findings suggest that specific *MED12* exon 2 mutations may also be involved, albeit rarely, in the development of colorectal tumours.

Leukaemias have been shown to develop in murines with retroviral insertions in *Med12* (Dave *et al*, 2009). A role for the CDK8 module/*MED12* in hematopoiesis has also been suggested in *Drosophila* and zebrafish, respectively (Gobert *et al*, 2010; Keightley *et al*, 2011). High *MED12* expression level has been observed in acute myeloid leukaemia and acute lymphoid leukaemia compared with other tumour types in the GeneSapiens database (http://www.genesapiens.org/) (Kilpinen *et al*, 2008). In line with a previous study by Je *et al*, no *MED12* exon 2 mutations in any haematological malignancies were detected (Je *et al*, 2012).

Taken together, screening of 1158 samples representing various tumour types revealed five *MED12* exon 2 mutations; three in uterine leiomyosarcomas and two in CRC samples. Three mutations in the confirmed uterine leiomyosarcomas indicate that these tumours may have developed through a leiomyoma precursor. Supported by the observations by Je *et al* and The Cancer Genome Atlas Network, we also suggest that *MED12* exon 2 mutations may contribute, albeit rarely, to CRC tumorigenesis (Je *et al*, 2012; The Cancer Genome Atlas Network, 2012). Interestingly, Barbieri *et al* recently reported recurrent *MED12* mutations affecting codon 1224 in 5 out of 111 (4.5%) prostate adenocarcinomas studied (Barbieri *et al*, 2012). It remains to be seen whether additional mutations in other parts of this large gene have a role in the development of various tumour types. The high *MED12* exon 2 mutation frequency observed in uterine leiomyomas seem to associate with the location of the tumours in the uterus and also with their benign nature. The mechanistic details and the affected molecular pathways through which these extremely specific mutations promote tumorigenesis need to be unravelled at the molecular level utilising, for example, gene expression analyses, mouse models, and *in vitro* functional experiments. It is likely they alter a very specific function of the MED12 protein providing the cells with a growth advantage especially in the uterus.

## Figures and Tables

**Table 1:** Tumours included in the *MED12* exon 2 mutation screening

**Tumour type**	***N***	**Sample type**	**Samples from**
*Mesenchymal*			
Uterine leiomyosarcoma	39	FFPE	PH/CFCH
Early onset (dg⩽45 years)	27		PH
Unselected	12		CFCH
Sarcoma	104	Fresh frozen	PH
Soft tissue sarcoma (including two ULMS)	83		
Bone sarcoma	21		
Gastrointestinal stromal tumour	12	FFPE	CFCH
Extrauterine leiomyoma	42	FFPE	CFCH/PH
Endometrial polyp	54	FFPE	PH
Lipoma	35	FFPE	CFCH
			
*Oestrogen*–*progesterone dependent*			
Ovarian carcinoma	122	FFPE/fresh frozen	PH
Clear cell	39		
Serous	44		
Mucinous	10		
Endometrioid	10		
NOS	19		
Breast cancer	94	Fresh frozen	OGH/PH
Ductal	68		
Lobular	14		
Medullary	4		
Other	8		
			
*Haematological malignancies*			
Acute myeloid leukaemia	131	Fresh/fresh frozen	AAUH/MSKCC
Acute lymphoid leukaemia^a^	37	Fresh frozen	AAUH
Myeloproliferative neoplasm	96	Fresh/fresh frozen	MSKCC
Polycythemia vera	48		
Essential thrombocytosis	48		
			
*Abnormal Wnt-signalling associated*			
Colorectal cancer	392	Fresh frozen	FCH

Abbreviations: AAUH=Department of Hematology at Aarhus University Hospital; CFCH=Central Finland Central Hospital; FCH=Finnish Central Hospitals; FFPE=formalin-fixed paraffin-embedded; MSKCC=Memorial Sloan-Kettering Cancer Center; NOS=not otherwise specified; OGH=Department of Obstetrics and Gynecology at Helsinki University Central Hospital; PH=Department of Pathology at Helsinki University Central Hospital; ULMS=uterine leiomyosarcoma.

a T-cell origin.
