# Supplementary material for: Somatic MED12 mutations in uterine leiomyosarcoma and colorectal cancer
Source: Br J Cancer. 2012 Sep 20;107(10):1761–5. doi: 10.1038/bjc.2012.428 (PMC3493861; doi:10.1038/bjc.2012.428)
Supplement: Supplementary Information [file bjc2012428x1.pdf]

SUPPLEMENTARY INFORMATION FOR

Somatic *MED12* mutations in uterine leiomyosarcoma and colorectal cancer

Kati Kämpjärvi, Netta Mäkinen, Outi Kilpivaara, Johanna Arola, Hanna-Riikka Heinonen, Jan Böhm, Omar Abdel-Wahab, Heli J. Lehtonen, Liisa Pelttari, Miika Mehine, Heinrich Schrewe, Heli Nevanlinna, Ross L. Levine, Peter Hokland, Tom Böhling, Jukka-Pekka Mecklin, Ralf Bützow, Lauri A. Aaltonen, and Pia Vahteristo*

*To whom correspondence should be addressed. E-mail: pia.vahteristo@helsinki.fi

This PDF file includes:

Supplementary Text
Supplementary Table 1
Supplementary Table 2
Supplementary Table 3
Supplementary Figure 1
Supplementary Figure 2
Supplementary References

Supplementary Text

Histopathology of uterine leiomyosarcomas

We have previously collected a series of 67 archival uterine leiomyosarcoma samples representing a population-based material of early onset uterine leiomyosarcoma cases (dg $\leq$ 45 years) identified between years 1981 and 2003 through a systematic search from the Finnish Cancer Registry [Ylisaukko-oja *et al*, 2006]. The diagnostic criteria for uterine leiomyosarcomas have, however, changed since the sample collection. Hematoxylin-eosin (HE)-stained sections were available from 59 samples, and they were reviewed by an expert in gynecological pathology (JA). The tumors were classified according to the new WHO criteria [Tavassoli and Devilee, 2003]. The number of mitotic figures per 10 high power fields, the degree of cellularity (normal, cellular, highly cellular), severity of cytological atypia (1-3), and the presence of necrosis was recorded for each case.

After reviewing the samples, only 27 out of 59 tumors were classified as uterine leiomyosarcomas. Rest of the tumors, altogether 32 lesions, represented various leiomyoma subtypes including one common, eight atypical, four cellular, one epithelioid, and 18 mitotically active leiomyomas. See Supplementary Table 1 for details.

Additional 12 uterine leiomyosarcoma samples were collected for this study at the Central Finland Central Hospital, Jyväskylä, Finland. These samples were evaluated by a pathologist (JB), after which the samples were anonymized. Also two out of 83 soft tissue sarcomas from the Department of Pathology at Helsinki University Central Hospital, Helsinki, Finland were confirmed as metastases of uterine leiomyosarcomas after evaluation by a pathologist (TB). Thus, altogether 41 uterine leiomyosarcomas were included to the study.

DNA extraction

Genomic DNA was extracted from the archival FFPE tissue samples either with a standard method (proteinase K digestion followed by phenol-chloroform extraction, more detailed instructions are available upon request) or with NucleoSpin® FFPE RNA/DNA Kit (Macherey-Nagel, Düren, Germany). Genomic DNA from the fresh frozen tissue samples, excluding hematological neoplasms, was extracted with a conventional non-enzymatic method [Lahiri and Nurnberger, 1991] or with FastDNA®-kit (MP Biomedicals LLC (Illkrich, France). DNeasy® Blood and Tissue Kit (Qiagen, Hilden, Germany) was used to purify total DNA from ALL- and MPN-samples, as well as from those AML-samples that were sequenced at the Memorial Sloan-Kettering Cancer Center (MSKCC). Genomic DNA of AML-samples obtained from Aarhus University Hospital (AAUH) was extracted with MagNA Pure LC DNA Isolation Kit I (Roche Applied Science, Penzberg, Germany).

***MED12* exon 2 mutation screening**

MED12 exon 2 mutation status was determined by direct sequencing. For the fresh frozen tissue samples, except breast and ovarian carcinomas, the previously published oligonucleotide primers were used [Mäkinen *et al*, 2011]. The rest of the samples were screened using primers which amplify the mutation hotspots and all the observed insertions and deletions in the *MED12* exon 2 region. These primer sequences in the 5' to 3' direction are GCCCTTTCACCTTGTTCCTT (forward) and AAGCTGACGTTCTTGGCACT (reverse). PCR conditions are available upon request. Sequencing was performed on an ABI3730 Automatic DNA Sequencer (Applied Biosystems at FIMM Genome and Technology Centre, Finland) according to manufacturer's instructions. The sequences were analyzed both manually and with Mutation Surveyor-software (Softgenetics, State College, PA, USA).

Supplementary Table 1. Histopathological information of the 59 tumors originally diagnosed as early-onset uterine leiomyosarcomas. After re-evaluation, 27 samples fulfilled the revised WHO-criteria for leiomyosarcoma, and were used in the *MED12* screening. Out of the 27 uterine leiomyosarcomas two were *MED12* mutation positive and are in bold.

Tumor sample	Diagnosis	Mitotic index /10HPF	Atypia	Cellularity	Necrosis
LM_1.1T	Leiomyosarcoma	5-9	3	+	+
LM_2.1T	Cellular	5-9	1	++	-
LM_3.1T	Mitotically active	5-9	1	+	-
LM_4.1T	Atypical	5-9	3	+	-
LM_5.1T	Atypical	<5	3	-	-
LM_6.1T	Leiomyosarcoma	5-9	3	+	+
LM_7.1T	Leiomyosarcoma	10-20	3	+	-
LM_8.1T	Atypical	5-9	3	+	-
LM_9.1T	Leiomyosarcoma	>20	3	+	+
LM_10.1T	Mitotically active	5-9	2	+	-
LM_11.1T	Mitotically active	10-20	1	++	-
LM_13.1T	Mitotically active	5-9	1	+	-
LM_14.1T	Leiomyosarcoma	5-9	1	+	+
LM_16_1T	Atypical	<5	2-3	+	-
LM_17.1T	Leiomyosarcoma	>10	2	+	+
LM_19.1T	Leiomyosarcoma	10-20	2	++	+
LM_20.1T	Mitotically active	5-9	2	+	-
LM_21.1T	Mitotically active	5-9	2	+	-
LM_22.1T	Mitotically active	10-20	1	++	-
LM_23.1T	Leiomyosarcoma	10-20	1	++	+
LM_25.1T	Atypical	5-9	2	++	-
LM_28.1T	Mitotically active	5-9	1	++	-
LM_29.1T	Cellular	<5	1	++	-
LM_30.1T	Leiomyosarcoma	10-20	3	++	-
LM_31.1T	Leiomyosarcoma	10-20	3	+	+
LM_32.1T	Atypical	5-9	2	+	-
LM_33.1T	Leiomyosarcoma	5-9	2	++	+
LM_34.1T	Leiomyosarcoma	5-9	3	++	+
LM_36.1T	Leiomyosarcoma	10-20	2	++	+
LM_38.1T	Atypical	5-9	3	+	-
LM_40.1T	Mitotically active	5-9	1	++	-
LM_41.1T	Cellular	5-9	1	++	-
LM_43.1T	Mitotically active	5-9	2	+	-
LM_45.1T	Leiomyosarcoma	10-20	2	+	-
LM_47.1T	Mitotically active	5-9	1	+	-
LM_48.1T	Mitotically active	10-20	1	+	-
LM_50.1T	Atypical	5-9	3	+	-
LM_51.1T	Epithelioid	<5	2	+	-
LM_52.1T	Leiomyosarcoma	10-20	3	++	+

Tumor sample	Diagnosis	Mitotic index /10HPF	Atypia	Cellularity	Necrosis
**LM_53.1T**	**Leiomyosarcoma**	**10-20**	**1**	**++**	**+**
LM_54.1T	Common leiomyoma	<5	1	+	-
LM_55.1T	Cellular	<5	1	++	-
LM_57.1T	Mitotically active	5-9	1	+	-
LM_58.1T	Leiomyosarcoma	5-9	2	+	+
LM_59.1T	Mitotically active	10-20	1	+	-
LM_61.1T	Leiomyosarcoma	>20	3	+	+
**LM_62.1T**	**Leiomyosarcoma**	**5-9**	**2**	**+**	**+**
LM_64.1T	Mitotically active	>20	1	+	-
LM_65.1T	Leiomyosarcoma	10-20	3	++	-
LM_66.1T	Mitotically active	5-10	1	+	-
LM_67.1T	Leiomyosarcoma	>20	3	+	+
LM_69.1T	Leiomyosarcoma	>20	3	++	+
LM_71.1T	Leiomyosarcoma	10-20	2	++	-
LM_74.1T	Mitotically active	10-20	1	+	-
LM_75.1T	Leiomyosarcoma	5-9	1	++	-
LM_76.1T	Leiomyosarcoma	5-9	2	++	+
LM_79.1T	Mitotically active	5-9	2	+	-
LM_80.1T	Leiomyosarcoma	>20	3	+	+
LM_81.1T	Leiomyosarcoma	5-9	3	++	+

Supplementary Table 2. Forty-two anonymous extrauterine leiomyomas included in the *MED12* exon 2 mutation screening.

Sample number	Site of the leiomyoma	*MED12* exon 2 status
**Anonymous extrauterine leiomyoma samples collected at Central Finland Central Hospital**		
KSKS1	colon	wt
KSKS2	colon	wt
KSKS3	colon	wt
KSKS4	colon	wt
KSKS5	colon	wt
KSKS6	colon	wt
KSKS7	rectum	wt
KSKS8	colon	wt
KSKS9	skin (scrotum)	wt
KSKS10	soft tissue (wrist)	wt
KSKS11	skin (scrotum)	wt
KSKS12	skin (armpit)	wt
KSKS13	rectum	wt
KSKS14	soft tissue (wrist)	wt
KSKS15	rectum	wt
KSKS16	epididymis	wt
KSKS17	soft tissue (knee)	wt
KSKS18	skin	wt
KSKS19	stomach (cardia)	wt
KSKS21	soft tissue (leg)	wt
KSKS22	kidney	wt
KSKS23	soft tissue (sole)	wt
KSKS24	colon	wt
KSKS26	colon	wt
KSKS27	colon	wt
KSKS28	soft tissue (knee)	wt
KSKS29	stomach	wt
KSKS30	skin (shoulder)	wt
KSKS31	abdominal wall	wt
KSKS32	soft tissue (Achilles tendon)	wt
**Anonymous skin leiomyoma samples collected at Helsinki University Central Hospital**		
IL1	skin	wt
IL2	skin	wt
IL3	skin	wt
IL4	skin	wt
IL5	skin	wt
IL6	skin	wt
IL7	skin	wt
IL8	skin	wt
IL9	skin	wt
IL10	skin*	wt
IL11	skin	wt
IL12	skin	wt

*FH deficient

Supplementary Table 3. Observed *MED12* exon 2 mutations.

Sample ID	Tumor type	Nucleotide change	Amino acid change
LM_62.1T	Uterine leiomyosarcoma	c.130G>A	p.Gly44Ser
LM_53.1T	Uterine leiomyosarcoma	c.115_116ins21	p.Ala38_Leu39insSerHisAspGluLeu ThrAla
GE05-129	Uterine leiomyosarcoma (metastasis)	c.104_106del3	p.Glu35_Leu36delinsVal
c834.1T	Colorectal cancer	c.130G>T	p.Gly44Cys
c43.1T	Colorectal cancer	c.200C>T	p.Ala67Val

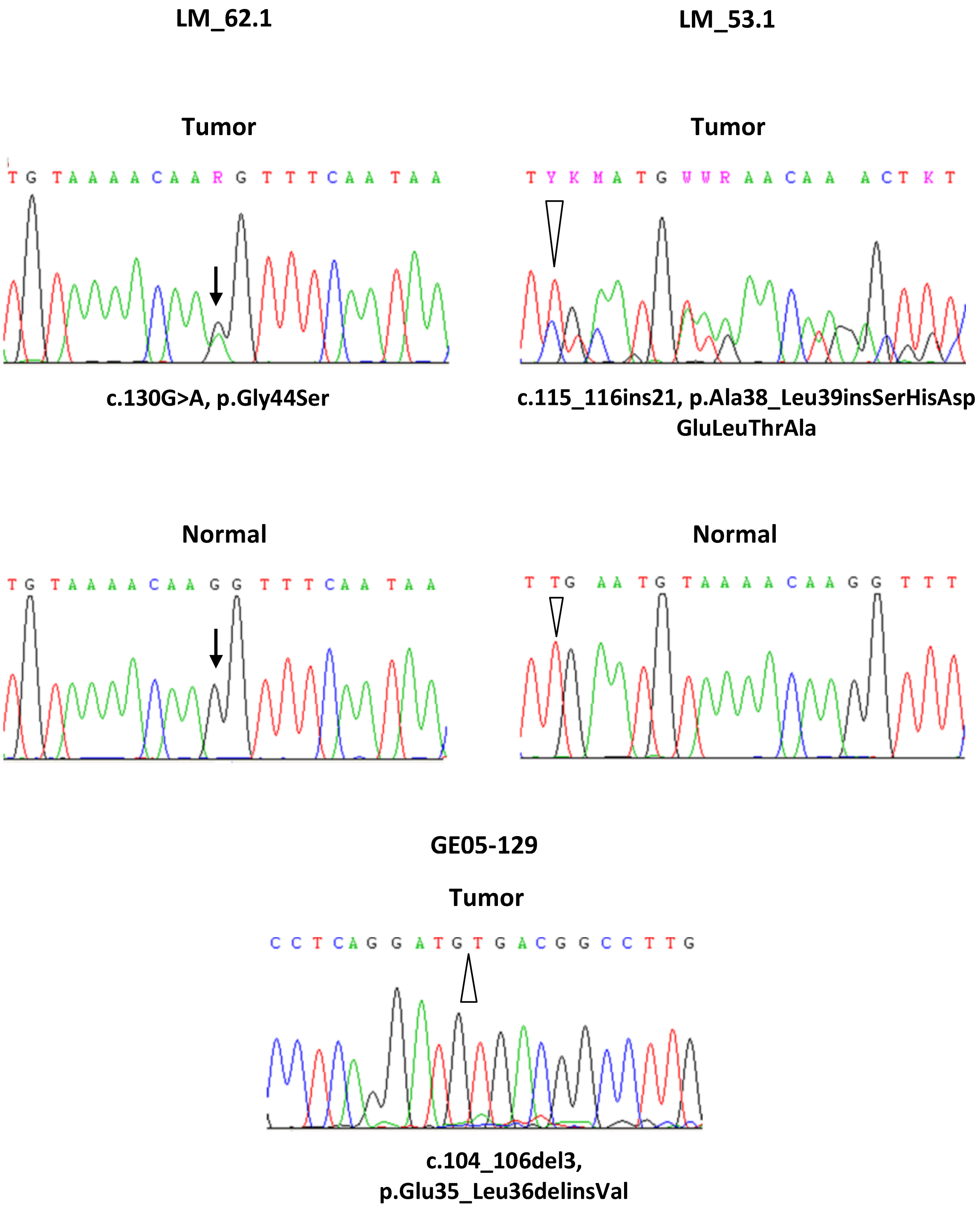

Supplementary Figure 1. Sequence chromatograms of three *MED12* exon 2 mutations detected in uterine leiomyosarcomas. From LM_62.1 and LM_53.1, sequences of both tumor and normal samples are shown.

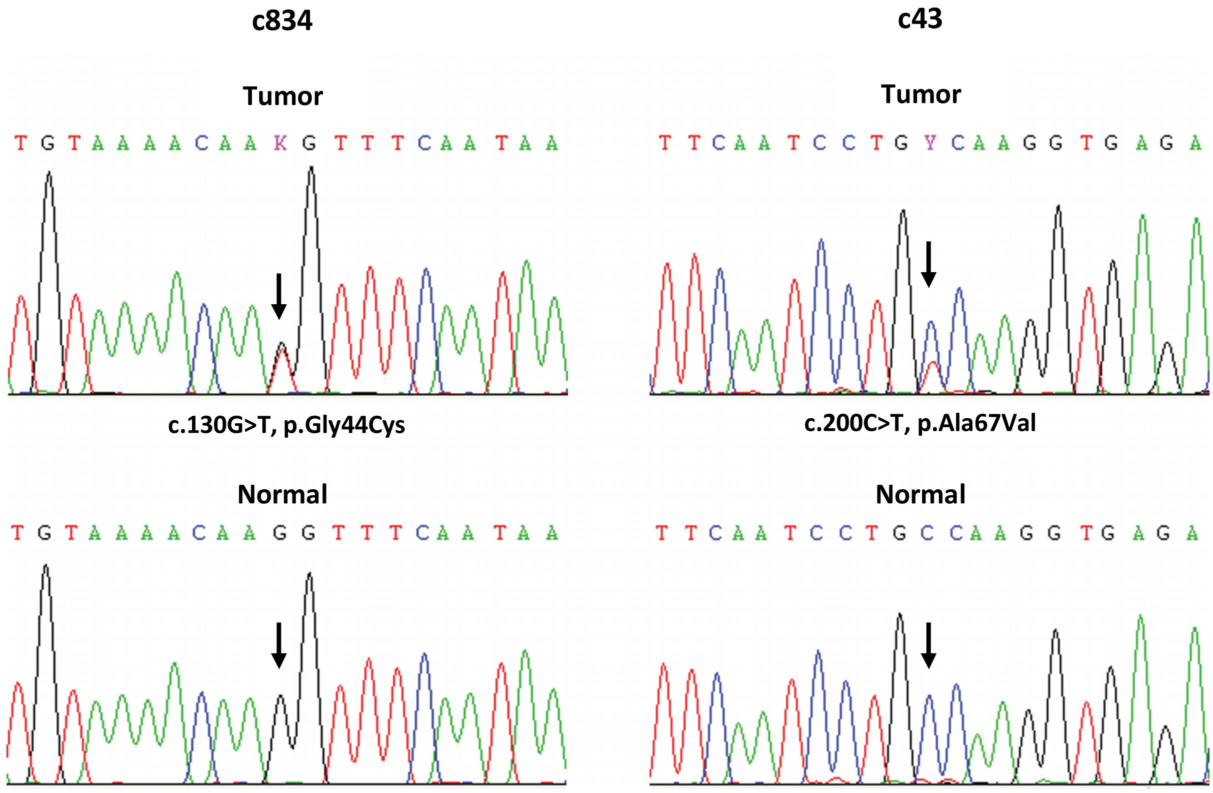

Supplementary Figure 2. Sequence chromatograms of two somatic *MED12* exon 2 mutations observed in CRC samples. Sequences of both tumor and normal samples are shown.

Supplementary References

Lahiri DK and Nurnberger JI Jr (1991) A rapid non-entsymatic method for the preparation of HMW DNA form blood for RFLP studies. *Nucleic Acids Res* **19**:5444

Mäkinen N, Mehine M, Tolvanen J, Kaasinen E, Li Y, Lehtonen HJ, Gentile M, Yan J, Enge M, Taipale M, Aavikko M, Katainen R, Virolainen E, Böhling T, Koski TA, Launonen V, Sjöberg J, Taipale J, Vahteristo P, Aaltonen LA (2011) MED12, the mediator complex subunit 12 gene, is mutated at high frequency in uterine leiomyomas. *Science* **334**: 252-255, doi:10.1126/science.1208930

Tavassoli FA and Devilee P (eds) (2003) World Health Organization Classification of Tumours. Pathology and Genetics of the Breast and Female Genital Organs. IARC Press: Lyon

Ylisaukko-oja SK, Kiuru M, Lehtonen HJ, Lehtonen R, Pukkala E, Arola J, Launonen V, Aaltonen LA (2006) Analysis of fumarate hydratase mutations in a population-based series of early onset uterine leiomyosarcoma patients. *Int J Cancer* **119**: 283-287, doi:10.1002/ijc.21798
